# Disease transmission and control modelling at the science–policy interface

**DOI:** 10.1098/rsfs.2021.0013

**Published:** 2021-10-12

**Authors:** Ruth McCabe, Christl A. Donnelly

**Affiliations:** ^1^ Department of Statistics, University of Oxford, 24–29 St Giles', OX1 3LB, Oxford, UK; ^2^ NIHR Health Protection Research Unit in Emerging and Zoonotic Infections, UK; ^3^ MRC Centre for Global Infectious Disease Analysis, Department of Infectious Disease Epidemiology, Imperial College London, London, UK

**Keywords:** COVID-19, transmission modelling, decision-making, science communication, non-pharmaceutical interventions, United Kingdom, public health policy

## Abstract

The coronavirus disease 2019 (COVID-19) pandemic has disrupted the lives of billions across the world. Mathematical modelling has been a key tool deployed throughout the pandemic to explore the potential public health impact of an unmitigated epidemic. The results of such studies have informed governments' decisions to implement non-pharmaceutical interventions to control the spread of the virus. In this article, we explore the complex relationships between models, decision-making, the media and the public during the COVID-19 pandemic in the United Kingdom of Great Britain and Northern Ireland (UK). Doing so not only provides an important historical context of COVID-19 modelling and how it has shaped the UK response, but as the pandemic continues and looking towards future pandemic preparedness, understanding these relationships and how they might be improved is critical. As such, we have synthesized information gathered via three methods: a survey to publicly list attendees of the Scientific Advisory Group for Emergencies, the Scientific Pandemic Influenza Group on Modelling and other comparable advisory bodies, interviews with science communication experts and former scientific advisors, and reviewing some of the key COVID-19 modelling literature from 2020. Our research highlights the desire for increased bidirectional communication between modellers, decision-makers and the public, as well as the need to convey uncertainty inherent in transmission models in a clear manner. These aspects should be considered carefully ahead of the next emergency response.

## 1. Background

Since the discovery of the novel coronavirus SARS-CoV-2 in late 2019, the lives of billions of people across the planet have been substantially disrupted. This highly transmissible virus, which causes coronavirus disease 2019 (COVID-19), has threatened the stability of healthcare systems globally with the official death count sitting at over 3.8 million people as of 15 June 2021 [1].

Mathematical models of transmission dynamics are commonly used to understand the dynamics and potential impacts of infectious disease outbreaks. As the initial epidemic unfolded in Wuhan in early 2020, scientists sought to understand rapidly the key epidemiological characteristics of the virus, such as the serial interval distribution and infection fatality ratio, in order to parametrize such models [2–4]. This then allowed the exploration of urgent public health questions, for example: how many people have been infected with the virus? Might the unmitigated spread of the virus overwhelm healthcare systems? [5,6] The results of such studies are one piece of evidence used to inform governments' decisions to take unprecedented actions to control the spread of the virus in the form of stringent non-pharmaceutical interventions (NPIs). As well as reducing transmission of the virus, these measures have drastically disrupted the normal functioning of society with no section of the population left unaffected by the pandemic and its control.

In the light of the unprecedented pandemic situation that we have collectively experienced, we have sought to explore the complex relationships between models, decision-making, the media and the public during the COVID-19 pandemic in the United Kingdom of Great Britain and Northern Ireland (UK). Doing so not only provides an important historical context of COVID-19 modelling and how it has shaped the UK response, but as the pandemic continues and looking towards future pandemic preparedness, understanding these relationships and how they might be improved is critical. As such, we have synthesized information gathered via three methods, encompassing both original, primary data gathered by the authors in addition to some key modelling literature. Combining these complementary sources gives a rich and holistic insight into the utility, diversity, development and influence of modelling in policy in a more detailed way than considering one of these sources in isolation. First, we considered some of the most prominent modelling studies internationally and then focus on specific modelling studies conducted in the UK, the results of which were presented to scientific advisory groups before the introduction of the first national ‘lockdown’ in March 2020. Second, we surveyed individuals publicly listed as members, or having attended a meeting, of the Scientific Advisory Group for Emergencies (SAGE), the Scientific Pandemic Influenza Group on Modelling (SPI-M) and other comparable advisory bodies about the development and role of modelling in COVID-19 decision-making. Third, we conducted interviews with individuals including science communication experts and former scientific advisors in order to understand further the interplay between these communities, particularly in comparison to previous health emergencies.

## 2. Methods

### 2.1. Literature review of COVID-19 modelling studies in 2020

Since the beginning of the pandemic, an extremely large number of studies involving mathematical modelling, restricted in this review to compartmental or individual-based models, of the transmission and control of SARS-CoV-2 have been published. While many make important contributions to the scientific understanding of the spread and control of the virus, we have sought to identify and synthesize a subset of key influential studies published in 2020. Therefore, this review does not synthesize all of the COVID-19 modelling literature.

We searched Web of Science (WoS) for (COVID-19 OR SARS-CoV-2 OR ‘coronavirus disease’) AND (model* OR simulat* OR impact) AND (transm* OR ‘non-pharmaceutical intervention’) on 15 December 2020. The results of this search were ordered according to WoS citation count and the 100 most cited papers were downloaded. Due to the rapidly evolving public health situation, many scientists published influential research as preprints to ensure this important information was publicly available without delay. Therefore, we also searched Google Scholar (GS) for (model COVID-19 OR SARS-CoV-2 OR ‘coronavirus disease’ OR ‘non-pharmaceutical intervention’ simulat OR impact OR transm). The restrictive nature of GS did not allow for an identical search to WoS, and we found that changing the order of search terms affected the search results. Searches in GS returned 1000 results and are presented according to a bespoke algorithm using undeclared relevancy criteria. These 1000 results and their corresponding GS citation counts were extracted on 15 December 2020.

**Figure 1.**
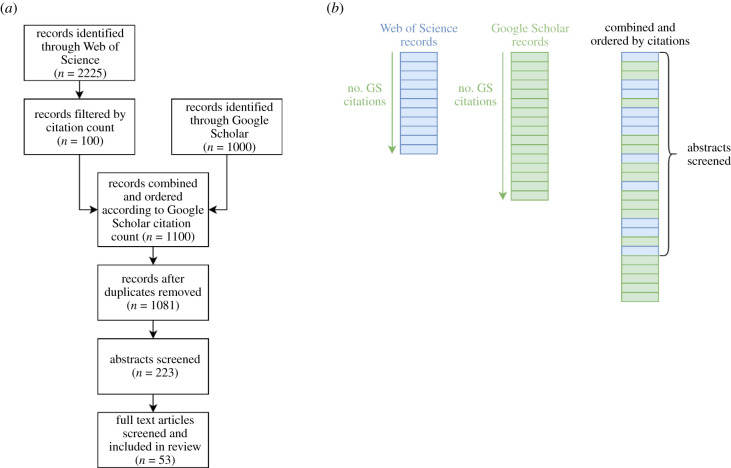
Overview of literature review search methodology. (*a*) Schematic diagram illustrating the process in which articles were identified and screened. (*b*) Illustration of how records from WoS and GS were combined and the number of abstracts that were screened was determined.

WoS citation counts encompass only those citations from other papers within the WoS database. Therefore, the GS citation count of a peer-reviewed paper often differs to WoS citation count due to the broader inclusion criteria. To combine the results of both searches, GS citation counts for each of the WoS papers were extracted on 15 December 2020. The two groups of studies were then combined, duplicates were removed, and the studies were ordered according to their GS citation count. Using this list, the abstracts were sequentially screened until all 100 WoS studies had been considered. Systematic reviews and publications without mathematical models of SARS-CoV-2 transmission and disease progression as defined above were excluded from the review. Figure 1 summarizes this process.

### 2.2. Survey to attendees of the Scientific Advisory Group for Emergencies, the Scientific Pandemic Influenza Group on Modelling and other comparable advisory committees

In order to further understand the development and role of modelling in COVID-19 decision-making, a short survey of eight multiple-choice and two longer-answer questions was sent to individuals publicly listed as being a member of, or having attended a meeting of, the SAGE, SPI-M or the Scottish Government COVID-19 Advisory Group during the pandemic [7–9]. In addition, a small number of individuals involved in similar committees overseas were also included. This study has approval from the Medical Sciences Interdivisional Research Ethics Committee at the University of Oxford with Ethics Approval Reference R74566/RE001. The participant information sheet (PIS) is included in the electronic supplementary material, appendix S1.

The survey was produced on Microsoft Forms and contained the questions set out in the electronic supplementary material, appendix, table S1. After each multiple-choice question, participants were invited to provide any further details which they consider relevant to the question and/or their answer to said question via an optional open-ended question box. Participants were sent a link over email along with the PIS. Responses were gathered between 25 January 2021 and 30 March 2021. Answers to all multiple-choice questions remain anonymous. Participants were explicitly informed that any written answers provided, either via the open-ended questions or follow-up to multiple-choice questions, may be quoted in the article and attributed to them under one of three levels of anonymity: full anonymity, job title only, full name and job title (electronic supplementary material, appendix, table S1, question 18). Participants that have been quoted were notified of and consented to the exact wording used in the manuscript ahead of publication.

### 2.3. Interviews with science communication experts and former scientific advisers

To further examine the interplay between scientists, decision-makers, the media and the public with respect to modelling throughout the pandemic, individuals including science communication experts, former scientific advisers and policy engagement officers were invited to participate in interviews. The selection was based on the individual's expertise in the interface between science, policy and media and for which an email address could be obtained. This study has approval from the Medical Sciences Interdivisional Research Ethics Committee at the University of Oxford with Ethics Approval Reference R74566/RE001. The PIS can be found in the electronic supplementary material, appendix S3.

The interviewer prepared discussion points and questions ahead of the interview, which were shared with the interviewee upon request. Discussion points broadly covered: the responsibilities and opportunities of an individual in their role in the pandemic; their experience of the utility, or lack of, mathematical modelling throughout the pandemic; how communication between communities of interest could be improved and how the COVID-19 pandemic compared to previous health emergencies they have been involved in. The interviewer allowed for a natural flow of conversation wherever possible and encouraged interviewees to shape the conversation in the way that they desired in order to avoid influencing points raised by interviewees.

All interviews except for one were conducted virtually, with interviews lasting between 20 min and 1 h depending on the availability of the interviewee.

## 3. Results

### 3.1. Primary data collection

**Figure 2.**
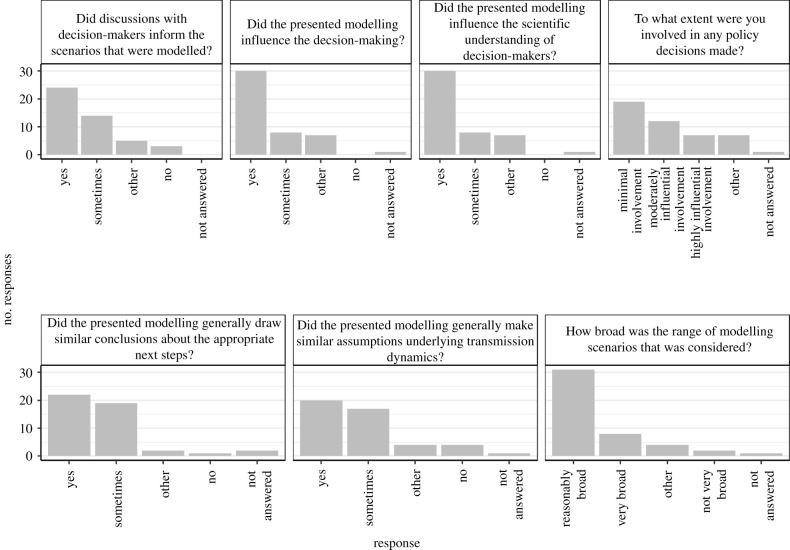
Results of the multiple-choice questions in the survey to attendees of SAGE, SPI-M and other comparable advisory bodies.

A total of 189 individuals were identified as either being a member of or having attended a meeting of SAGE, SPI-M or other comparable advisory groups as of January 2021. Of these, we found contact information online for 174 individuals who were subsequently invited to participate in the survey. We received a total of 46 responses (26% of those emailed; 24% of those identified), with most respondents participating anonymously. Figure 2 and electronic supplementary material, appendix table S1 present the results of the multiple-choice questions, the implications of which are discussed throughout the remainder of the article.

A total of 13 individuals were identified as suitable for interview based on the selection criteria. We conducted 11 interviews (85% of those contacted), the details of which are summarized in electronic supplementary material, appendix table S2.

### 3.2. Mathematical models for outbreak response

The transmission of infectious diseases is a complex process, the dynamics of which are driven by a combination of biological, sociological and environmental factors [10]. Scientists often use mathematical models to simplify complex real-world transmission and disease progression processes by splitting the population into distinct compartments from pre-infection to recovery or death, with flow through the model governed by epidemiological parameters estimated from a variety of data sources [11]. These models inherently require assumptions so that the process can be quantified and used to explore various questions (which may or not be directly of relevance to decision-makers).

While many models used in decision-making appear to be focussed primarily on providing epidemic projections based on certain assumptions or scenarios, models can be used far beyond this purpose: to understand a previous outbreak, to test the reliability of assumptions and to unravel the drivers of transmission. Additionally, studies measuring key parameters, such as the household attack rate, can inform decision-makers directly as well as through their estimates being used in transmission models.

Professor Sir Charles Godfray, Director of the Oxford Martin School, noted that the exploration of a situation as complex as an epidemic in which the future trajectory is unknown requires modelling: ‘There is no alternative’. This is reinforced by Professor Jason Leitch, National Clinical Director for the Scottish Government, who added that modelling ‘is crucial’.

### 3.3. The diversity of COVID-19 modelling

The WoS search returned 2225 papers in total of which the 100 most cited were extracted. The citation counts of these papers were (with one exception) greater in GS, with the order of most cited to least cited generally preserved. Nineteen duplicates were removed. From the remaining 1081 papers, the abstracts of the 223 most cited (thus encompassing the 100 most cited WoS studies) were screened. Of these, 53 met the inclusion criteria thus were included in the review (figure 1) [5,6,12–62].

Dr Sabine van Elsland, External Relationships & Communications Manager at the Medical Research Council Centre for Global Infectious Disease Analysis (MRC GIDA) at Imperial College London, noted her pride in the way the academic community came together to understand the characteristics of SARS-CoV-2. This is exemplified by the breadth of studies included in our review, many of which were published within just weeks of the beginning of the outbreak in Wuhan.

Models included in the review broadly followed susceptible–infected–recovered (SIR) or susceptible–exposed–infected–recovered (SEIR) structures, but are diverse in their applications and adapted as required to meet the needs of specific investigations. A common example of this among the reviewed papers was the inclusion of compartments for individuals requiring hospitalization (15 studies [5,6,14,16,22,25,28,32,37,41,44–46,53,56]). Some researchers opted for more bespoke adaptations: Kucharski *et al*. [13] duplicated model compartments for the population in Wuhan and international travellers while Eikenberry *et al*. [28] split the population into compartments according to mask-wearing habits. The diversity spanned beyond model structure. For example, studies in our review examined outbreaks in different locations [6,15,18,22,29], the impact of factors such as age or asymptomatic infection on transmission [12,13,23,61], the use of NPIs as a control measure [5,17,28,37], or, often, a combination of multiple factors. In isolation, each study contributes an interesting analysis, but together they build a rich and wide-ranging scientific understanding of SARS-CoV-2 and options for its control.

Different data sources and the way model parameters were estimated varied more than basic model structure. Modellers examining the early outbreak in Wuhan had limited data with which to parametrize models, which often centred on relatively small numbers confirmed COVID-19 cases and even used biological characteristics of the severe acute respiratory syndrome coronavirus (SARS-CoV-1) [3,4,12–14,16,24,31,33,62]. More informative data fed into models as they became available, for example as the epidemic spread to other locations and more affected countries published their epidemiological data. This was explained by Professor Leitch, who noted the improvement of mathematical modelling over the course of the pandemic as more information had become available as well as people's understanding of it: ‘The modelling has gotten better because 14 months ago [March 2020] we didn't know what this virus was, it didn't have a name. So now we know a lot more about what it [the virus] does and how it behaves and so we end up with better modelling and better translation of modelling’. Nonetheless, 32 studies in our review reported data quality, uncertainty in epidemiological parameters and/or rapidly changing insights generated from data in the unfolding epidemics, as caveats of their analysis [5,6,13,14,18,20,21,23–25,26,27,29,31–35,39,40,41–44,46,49,54,55,59,60,61,62]. The final point was explicitly noted by SAGE with their published evidence [8] and was emphasized by many survey respondents and interviewees.

Throughout 2020, control of the virus centred on the implementation of NPIs, such as social distancing and school closures. As such, it is unsurprising that modelling their impact on metrics of interest, such as peak infections, peak hospitalizations and cumulative COVID-19-related deaths, was a central focus of most studies in this review (48 studies) [5,6,12–23,25–38,41–44,46–48,50–62]. Some also presented metrics relating to the effect of the intensity or timing of NPI implementation on reducing these metrics [5,6,16,21,37,46]. The specific interventions modelled varied substantially between studies: social distancing, quarantine and isolation of cases were among the most common, with 18 studies considering multiple interventions [5,6,12,17,18,20,23,25,26,29,30,33,37,44,46,53,56,58] and 11 studies considering generic reductions in transmission rather than a particular intervention [16,19,27,35,36,42,43,50,52,57,59]. These were modelled structurally in different ways, from altering transmission rate parameters or contact patterns, and implemented at different times, for example at fixed time points or triggered based on disease burden. This heterogeneity means that even within the same setting it is difficult to compare study results directly, but all studies in this review considering NPIs illustrated the potentially severe consequences of allowing a COVID-19 epidemic to progress without mitigation.

The authors of four studies self-reported their role in informing decision-making either in the UK or locally in the United States [5,6,45,47]. Weissman *et al*. [45] presented estimates of the demand for hospital resources from COVID-19 patients in Philadelphia under different assumptions of the epidemic doubling time. In contrast with the other three papers, Paltiel *et al*.'s [47] study did not focus on healthcare rather on the role of testing in preventing outbreaks on US college campuses. In addition to estimating the number of cases under different testing policies, they provided an analysis of the cost effectiveness of each scenario in relation to the number of tests and cases averted. Other studies in our review are likely to have been used in decision-making as well, but the authors not have declared this explicitly.

### 3.4. The COVID-19 pandemic and science advisory mechanisms in the United Kingdom of Great Britain and Northern Ireland

Within 1 year of recording the first confirmed case of COVID-19 on 30 January 2020, the UK had registered almost 4 million confirmed cases and nearly 110 000 deaths due to the virus [63,64]. This was in addition to the substantial economic toll, with a reduction in gross domestic product growth and an increase in unemployment [65,66]. At the time of revision (June 2021), since the government implemented the first national lockdown on 23 March 2020, most of the population have remained under some form of restrictions aimed at controlling the spread of the virus [67].

SAGE is the central advisory body responsible for providing ‘scientific and technical advice to support government decision-makers during emergencies’ [68]. Professor Leitch noted that if decision-makers were not being asked to make such difficult and complex decisions, there would not be a need for specialist advisers. An anonymous survey respondent underlined their role as an adviser: ‘Policy decisions are made by policymakers. Our job is to ensure they have the evidence.’. This was corroborated by multiple survey respondents, with 41% stating that they had ‘minimal involvement’ in any policy decisions, compared to just 15% who considered their involvement as ‘highly influential’ (figure 2). An overview of the science advisory mechanisms in the UK is given in [69].

**Figure 3.**
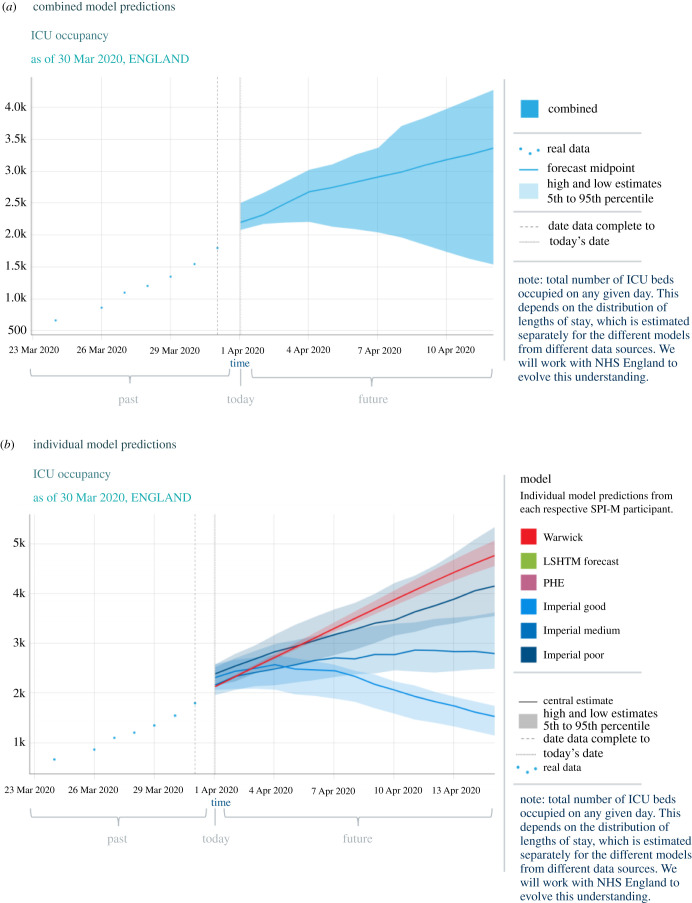
An example of modelling produced by SPI-M modelling groups in late March 2020 to provide short-term predictions of ICU occupancy in England in April 2020 [70]. (*a*) The individual modelling outputs, undertaken independently by different modelling groups, are combined to provide a wide range of possible trajectories for the epidemic in the following fortnight. (*b*) The outputs from the six individual model analyses which make up that presented in (*a*) are presented.

Scientific participants of SAGE are politically independent experts from a range of fields, which during the COVID-19 pandemic has included medicine, virology and epidemiology [7]. SAGE synthesizes scientific evidence pertinent to the threat posed by SARS-CoV-2 from a variety of sources, of which modelling is only a subset as noted by multiple survey respondents and Dr Claire Craig, former Director of the Government Office for Science. Mathematical modelling of transmission dynamics and disease progression is the primary focus of SPI-M, a specialist advisory group consisting of politically independent modelling experts which reports to SAGE. Professor Godfray noted that he has been impressed by the way in which the modelling community in the UK has ‘stepped up to support decision-making’ throughout the pandemic. As stated by an anonymous survey respondent, ‘SAGE meets to consider the results of the various models and then to develop a consensus statement (for decision-makers) that summarizes what can be inferred from these’. Figure 3*a* shows an example of combined model outputs from different SPI-M modelling groups to forecast intensive care unit (ICU) occupancy in early April 2020, while figure 3*b* presents the individual model outputs that this combined output is comprised [70]. Although some survey respondents commented that they felt SAGE and SPI-M structures were ‘too large in their current form’, in a modelling-themed issue of *Philosophical Transactions of Royal Society B*, Dr Ellen Brooks-Pollock, Dr Leon Danon, Dr Thibaut Jombart and Dr Lorenzo Pellis note that ‘The way SPI-M-O operates evolved during 2020—starting with a small number of modellers and expanding to around 50 modellers regularly attending the weekly meetings. This plurality of opinion was key to generating robust and reliable advice.’ [69].

### 3.5. The use of transmission modelling in the UK COVID-19 pandemic response

Professor Sir John Beddington, former government Chief Scientific Adviser 2008–2013, drew on his experience of modelling during the H1N1 influenza pandemic in 2009. He explained that, in light of many unknown parameter values, scenario-based modelling is the most appropriate and informative analysis for scientists to conduct. However, he recognized that during this pandemic, the modelling community are fortunate to have nationwide surveys to estimate COVID-19 prevalence over time, which can act as an independent verification of the results generated via mathematical models. This was further emphasized by Professor Linda Bauld, Bruce and John Usher Chair of Public Health at the University of Edinburgh, who noted that ‘Like all science, modelling needs to be triangulated with other data sources and understood in the wider context’.

Two papers included in our review contained results of scenario-based analyses that were presented to SAGE prior to the first national lockdown in March 2020 [5,6]. Both explored the potential future impact of broadly similar NPIs on measures of epidemic magnitude such as the number of infections, the number of deaths and healthcare capacity, another key metric of COVID-19 control for the UK government [71]. In both cases, estimates of peak hospital bed demand from COVID-19 patients were presented alongside the corresponding length of time spent under NPI restrictions for a range of scenarios, highlighting the trade-offs inherent in such interventions. The nuances of the individual models, both in the differing assumptions and stochasticity arising in the model fitting process, mean that specific estimates from these studies are not directly comparable. Nonetheless, both studies fully aligned on their overall conclusions that without sufficient mitigation critical care resources would be overwhelmed at several orders of magnitudes above baseline capacity. As explained by Dr Marc Baguelin, a researcher at Imperial College London and London School of Hygiene and Tropical Medicine (LSHTM), ‘How fast models reach a conclusion differs but a clear majority of models are usually in favour of a specific decision/outcome’, a statement corroborated by other survey respondents.

At the time of revision (June 2021), the aforementioned themed issue of *Philosophical Transactions of the Royal Society B* entitled ‘Modelling that shaped the early COVID-19 pandemic response in the UK’ was published featuring 20 articles from various modelling groups regularly contributing to SPI-M during the pandemic [69]. In addition to further highlighting the diversity and applications of modelling in informing policy, the editors provided a timeline (January–July 2020) of when the work in each article was undertaken which illustrates the evolution of the focus of and data available to the contributing scientists over the first wave of the virus as mentioned in Results: the diversity of COVID-19 modelling. For example, the first article used a mathematical model to estimate the basic reproduction number, *R*_0_, in Wuhan (early 2020) [72]. Shortly after, scientists used parameters estimated using the outbreak in Wuhan to parameterize models to understand the potential spread of SARS-CoV-2 in England and Wales [73]. As the pandemic progressed, scientific understanding of the virus increased and more data became available, modelling was applied to consider more specific issues, for example, the risk and impact of outbreaks in care homes [74] and the implications of waning population immunity on transmission in the UK [75].

While the power of mathematical models is well understood, their influence on policy can be difficult to quantify [76]. Nonetheless, Scott Heald, Head of Profession for Statistics at Public Health Scotland, as well as Professor Leitch, highlighted the utility of models in planning for a range of policy options within the Scottish Government. For example, Heald noted that estimates of the number of cases have been used to inform the resources required for the test-and-trace programme in addition to hospital resources. It has been reported that ‘the ‘Scottish Government uses the publically available Imperial model as reported in their Report 13 [18] to help understand the COVID-19 epidemic in Scotland over the longer term and what the reproductive rate at a point in time (*R*_t_) is for Scotland.’. Above all, Heald underlined, similarly to Professor Beddington and Professor Leitch, that the rapidly changing situation meant that while no model could reliably predict exactly what will happen in the future, the results can be reliably used to demonstrate what will happen if sufficient action is not taken. As noted by Professor Leitch: ‘Modelling doesn't lend itself to binary choice, but it is part of the framework of information that we had to take into account’. These statements were further corroborated by several survey respondents, with one noting that models give ‘real decision advantage’.

### 3.6. Development of modelling for use in decision-making

Modelling is only a useful tool to decision-makers if it considers ‘realistic’ policy options, as noted by an anonymous survey respondent. Most survey respondents (85%) agreed that discussions with decision-makers at least sometimes informed the scenarios that were modelled and presented to SAGE and/or SPI-M (figure 2). One such respondent noted that this very much depends on what is being asked, adding that ‘Sometimes it is very constrained by scenario other times people are free to bring interesting output and analyses to the group’. Dr Craig noted that the availability of evidence and data can often frame policy discussions, likening this to lamp-posts illuminating distinct areas on, but not the entirety of, the ground (issues pertaining to the policy discussion). Nonetheless, when asked about scenarios covered at these committees, 82% of respondents categorized the range considered as at least ‘reasonably’ or ‘very’ broad (figure 2). This is illustrated by Dr Mike Tildesley, a researcher at the University of Warwick, who noted: ‘There have been a significant number of different modelling scenarios considered by SPI-M, from short- and long-term forecasting, to the effect of school re-opening, to the effect of Christmas relaxation, the impact of bubbles, transmission on university campuses, the impact of a circuit breaker lockdown etc.’, and echoed by Professor John Edmunds, a researcher at LSHTM, who stated ‘We have tried to cover the main policy options’. One respondent noted that while ‘it was always possible to be broader’, it is ‘important that scenarios were reasonable’.

Respondents also generally noted a degree of flexibility given to modellers in the underlying assumptions in their models. Dr Ellen Brooks-Pollock, a researcher at the University of Bristol, noted ‘SPI-M contributors have been encouraged to make their own and best assumptions based on the evidence available at the time’, while Billy Quilty, a researcher at LSHTM, added that ‘Requests were generally broad, with the specific scenarios and assumptions determined through conversations within our modelling group and with SPI-M/SAGE members’. Despite this breadth and flexibility, the majority of survey respondents (79%) believed that different models were generally based on similar assumptions (figure 2). This is exemplified through a comparison of the studies by Ferguson *et al*. [5] and Davies *et al*. [6].

### 3.7. Communication between modellers and decision-makers

When asked how communication between modellers and decision-makers could be improved, many noted the success of the system at present. According to Professor Edmunds: ‘I actually think that the structures, such as SPI-M and SAGE have worked very well’, with another, Dr Lorenzo Pellis, a researcher at the University of Manchester, adding ‘Obviously, the current state of things has evolved since the start of 2020, so if future health emergencies were starting from the current level of communication, it would be great’. Many respondents stated that while modelling scenarios were informed by decision-makers, these conversations were often conducted by a third party, i.e. the SPI-M secretariat, rather than between the two parties directly. Furthermore, many suggested that communication flowed more often from modellers to decision-makers than in the opposite direction. Another respondent would have preferred to ‘Have an actual conversation between the two parties, rather than a one-way road. Key priorities and questions should be discussed between policymakers and modellers as there is often a trade-off between what decision-makers want to know, and what is achievable given available data’. Dr Craig echoed that discussions between the two communities are important, particularly regarding real-time work as during the pandemic: ‘most of the time what you really want is discussions such that the questions scientists ask and answer are informed by the policymaking, but the policymaking questions is informed by an understanding of what the science is telling them and also what it's capable of doing’. The potential mutual benefits of two-way communication were echoed by many respondents, with some suggesting that decision-makers should attend SAGE and SPI-M meetings to ‘hear the nuances of the debates' and ‘to enable the modelling teams to communicate the impact of uncertainty upon modelling outcomes’.

Models of disease transmission are inherently complex and understanding the intricacies of how they work requires a substantial investment of time. William Pryor, Head of Policy Engagement at the University of Oxford, noted the mutual benefit to be derived from the more open exchange of evidence and expertise between decision-making and research communities. He also noted, however, that both needed to develop the skills required to combine the breadth of the former's knowledge and depth of the latter's. As noted by Professor Graham Medley, chair of SPI-M, modellers have the responsibility of ensuring that they develop as high-quality models as possible, but how these are used by decision-makers is out of their control. Another respondent touched upon this: ‘There are many more considerations in policy than just the science’, a sentiment echoed by Dr Craig and Professor Godfray in our interviews, as well as Sir Patrick Vallance, government Chief Scientific Adviser, in [77]. These differences in perspective can make communication challenging, especially during an emergency such as the COVID-19 pandemic, as explained by Pryor and also Allen *et al*. in their report for the International Network for Government Science Advice [78].

Communicating the uncertainty inherent in mathematical models to decision-makers was highlighted by Lord John Krebs, former Chairman of the UK Food Standards Agency, and Professor Beddington as a key area requiring improvement and confirmed by many survey respondents. One noted: ‘I think outputs appear to influence the thinking of decision-makers, though they are often too focused on a single scenario, rather than adequately incorporating the uncertainty inherent in such tasks’. As highlighted by our literature review and discussed in [79–82], the uncertainty in models ranges beyond measures of traditional statistical uncertainty: model parametrisation, the choice and structure of modelling frameworks and further assumptions about the drivers of transmission all introduce additional layers of uncertainty into models which are essential to communicate in the results. While these issues existed long before the discovery SARS-CoV-2, Professor Beddington noted that the issue of unknown parameter values is more prominent during the COVID-19 pandemic due to the novelty of the virus than it was during the H1N1 influenza pandemic which behaved similarly to other strains of influenza. In a seminar hosted by Turing-RSS Laboratory and Joint Biosecurity Centre in April 2021 [83], Professor Marc Lipsitch commented that equal emphasis should be placed on highlighting the assumptions underpinning the results as is given to the results themselves through the use of ‘if X then Y’ statements rather than focussing solely on the ‘then Y’ aspect.

Many survey respondents noted that both communities have a responsibility to use times between emergencies to improve preparedness. Professor Christine Middlemiss, UK Chief Veterinary Officer, drew on her experience of how a previous outbreak informed emergency responses since then: ‘Learn from lessons already learnt in the broader One Health space. (Foot and mouth disease) FMD 2001 provided a wealth of understanding on just how important and how to improve this communication, and we have invested significantly in it and evolved it’. In terms of modelling, Professor Medley noted: ‘During a pandemic there is no time to develop novel models and to inform policy—the timelines are very short. We really need a next generation of models to be developed in the coming years to better suit the next pandemic’. Professor Sir Jeremy Farrar, Director of the Wellcome Trust, highlighted the need to ‘Increase levels of knowledge, understanding, scientific literacy before any emergency. The work done in the 'non-crisis' times determines the outcomes in a crisis. Don't try and forge those links, those insights in a highly dynamic situation, you will always be behind the curve’.

### 3.8. Communication of modelling and decision-making to the public

The NPIs introduced to control the spread of SARS-CoV-2 have profoundly affected public life. According to an anonymous respondent: ‘Communication with policymakers hasn't really been a problem. Communication with the public a little more so’. Professor Bauld noted that when (frequently) asked about the data informing decision-making during media appearances, she must ‘always refer to the modelling, because in the basket of indicators it is not just [say] incidence, prevalence, test positivity, it is also modelling forward what hospital capacity could be’.

Like most decision-makers, the public are not experts in infectious disease modelling. Striking the balance between conveying the overarching results of a modelling study and the assumptions underpinning these results is challenging, as described by Robert Cuffe, Head of Statistics at the BBC, and echoed by Lord Krebs, Heald and Professor Leitch. Robert Cuffe also noted a transition away from reporting on individual modelling studies as occasionally observed at the beginning of the pandemic, with emphasis placed on measures such as case numbers instead. Although crucial to SAGE and SPI-M, Cuffe noted that ‘having multiple versions of the same number’ in itself is not of interest to the public. Professor Leitch and Professor Bauld corroborated this and noted that they communicate modelling to the public in general terms. For example, Professor Leitch would say that ‘cases will increase over the next week’, as opposed to ‘there will be [say] 127 new cases by next week’.

Several science communication experts, including Fiona Fox, Director of the Science Media Centre, Dr van Elsland, Professor Leitch and Professor Bauld, have been striving to make modelling studies more accessible to and better understood by the public, particularly throughout the COVID-19 pandemic. In particular, Professor Bauld spoke about the personal responsibility she has felt to use her 25 years' experience in public health to support the communication of scientific evidence used in decision-making in an understandable way to the public. Referring to modelling, she noted that her role is ‘to understand the main messages, communicate the uncertainty and then to know when to stop commenting and pass over to someone who understand the methods more than I do’.

Fox, Dr van Elsland and Professor Leitch noted a strong commitment from most journalists to ensuring that modelling results are communicated clearly and accurately. Dr van Elsland identified ‘transparency’ as among the most critical factors when communicating modelling results to the public, so that the caveats of the analysis are understood while ensuring that the results are taken seriously, as corroborated by [84]. She noted the importance of ‘being clear and open about what the models do and do not know’, a sentiment echoed by Fox, Lord Krebs, Dr Craig, Professor Godfray and Heald.

Professor Leitch stated that when communicating with the public during the pandemic, he is ‘not just trying to educate, but trying to get people to change their behaviour’ in line with government regulations, which, as an adviser, is an additional challenge. Since March 2020, he has had a regular slot on football show ‘Off the Ball’ on BBC Scotland [85], the ‘most listened to radio show in Scotland’ and with an audience that ‘doesn't listen to other radio shows', in which he answers COVID-19-related questions posed by listeners. Professor Leitch explained that: ‘[listeners] often see the pandemic through their own lens of, for example, their wedding or their football match and you have to try and make your answers relevant to that. What you try and do is the translate an individual situation into a message or lesson for a broader population because there's probably a hundred thousand people with the same challenge’.

Fox emphasized that modelling results should be communicated to the media and the public directly from the scientists themselves. This reinforces the distinction between the scientists (including modellers) and decision-makers, something which many survey respondents believe has been blurred during the current pandemic. Many scientists [79,86,87] have also raised this issue when criticizing the UK government's ‘following the science’ catchphrase during the pandemic. Professor Leitch stated that advisers ‘try to draw a distinction between the politician who is leading the briefing who has a particular job and the clinical advisers who have a different job and it is important to continuously redraw that distinction’, referring to the Scottish Government COVID-19 briefings. Fox and Lord Krebs both noted that this distinction is a key component of building public trust in both modelling and decision-making. Dr Craig added that even if there is a distinction between these communities, if the public does not perceive this to be the case then this perception could equally weaken their trust in the decision-making process.

## 4. Discussion

This article has sought to provide a broader perspective on the scope and use of mathematical modelling throughout the COVID-19 pandemic, particularly in informing policy in the UK. Our literature review, although not exhaustive, highlighted both the diverse applications of modelling in providing evidence on a wide range of scientific and policy-focussed issues and the pace at which researchers have worked since the emergence of SARS-CoV-2 in early 2020. The respondents to our survey and our interviewees confirmed that modelling informed decision-making at key stages of the unfolding COVID-19 epidemics in the UK, although it was a subset of the evidence considered by SAGE and decision-makers. Similarly, our interviewees highlighted a largely positive interest from the British media and public in understanding the mechanisms of mathematical modelling and how to interpret the results.

Not unexpectedly given the pressures on everyone, respondents and interviewees indicated that communication between communities could be improved, but multiple competing factors must be balanced. Many survey respondents suggested that more direct communication with decision-makers would benefit both parties, with some stating that decision-makers should attend SAGE meetings to hear the evidence debated. However, many survey respondents and interviewees sought to make the distinction between the roles of scientists and decision-makers clear. Throughout the pandemic, the degree of separation between these roles has varied [88]. For example, the news that unelected UK government officials had attended SAGE meetings proved controversial, with some questioning the independence of scientific advice [89,90]. On the contrary, minutes from the 58th SAGE meeting on 21 September 2020 [91] presented five options, including a circuit-breaker lockdown, for the ‘package of interventions [that] will need to be adopted to reverse this exponential rise in cases', which was not actioned by decision-makers at that time. Ultimately, understanding this distinction is crucial for the public, to whom the decision-makers are accountable.

Communication of uncertainty to non-modellers was also a key area identified by survey respondents and interviewees as requiring improvement. While uncertainty is inherent in all mathematical and statistical models, how to convey this clearly to non-technical audiences remains an active area of research [81,92], particularly in epidemiology [80]. Decision-makers need to judge the likelihood of different scenarios and their potential consequences ahead of implementing policy, especially policies with as wide-ranging impacts as national lockdowns. The rapidly evolving pandemic often necessitated fast decisions and communicating the intricacies underpinning the uncertainty in model results can be time-consuming. Methods for conveying uncertainty fall outside the scope of this article. However, some respondents alluded to improving the general scientific understanding of decision-makers, with Professor Charles Bangham of Imperial College London, noting the example of the Vice-President of Taiwan being a professor of epidemiology. Additionally, Professor Gabriel Leung, senior author of [21], was previously Under Secretary for Food and Health in Hong Kong [93]. However, such examples could further cloud the distinction between scientists and decision-makers. The communication of uncertainty to the public requires a careful balancing of transparency and interpretability. As noted by several survey respondents, this should be developed between emergencies to facilitate effective communication of uncertainty during emergency situations.

Vast quantities of new information about COVID-19 become available daily across the world. While this improves our collective understanding of the virus, this also puts unprecedented pressure on scientists and decision-makers. Professor Bauld noted a ‘huge pressure to get it [science communication] right in the face of changing evidence’. In a dynamic situation such as this one, it is critical that both parties acknowledge the implications of new evidence and use it to inform their actions. This also presents a challenge for the media in communicating this to the public in an understandable and coherent manner, but it is critical to any emergency response that society fosters an open-minded attitude to updated scientific evidence over time. The importance of doing so was underlined by Dr Pellis, Dr Ian Hall and Professor Medley in the BBC Documentary ‘Lockdown 1.0 — Following the science?’ [94], in which it is stated that revised estimates of the epidemic doubling time in March 2020 and the consequences this would have on hospital capacity were presented to SPI-M and subsequently passed onto SAGE, leading to ‘the cascade of full lockdown’. Although our knowledge of the virus is continuously improving, there are still a great many unknowns about the transmission of SARS-CoV-2. Professor Beddington highlighted the equal importance of culture in which scientists underline that a particular fact is unknown in addition to updating scientific evidence over time, something which he believes Professor Chris Whitty, Chief Medical Officer and Sir Patrick Vallance have done particularly well throughout the pandemic. The former was echoed by Fox, Tim Fielden and Professor Godfray in a discussion hosted by the Oxford Martin School in February 2021 [95].

Our literature review was only able to encompass a small subset of modelling studies released since the beginning of the pandemic, given the extremely large number published over the course of 2020. However, our review informed our wider discussion on modelling rather than constituting a systematic modelling review. In this special case of the COVID-19 pandemic, we included studies which are not peer reviewed. Many scientists have published preprints during the COVID-19 pandemic to ensure fast and unrestricted access to important new information in a rapidly evolving situation. Fox noted that while the ‘justification for this practice is clear’, she hopes that the scientific community return to the peer review system after the emergency phase of the pandemic is over.

Many of findings in this article are based on the experiences and opinions of relevant parties (based primarily in the UK), gathered by the authors. Although almost all those invited for the interview took part, most of those invited to take part in the survey did not. This is frequently the case in research studies, but caution is needed to avoid assuming that those who responded are representative of those who did not, or that these findings are applicable to settings outside of the UK. While we intended for our survey questions to be as open-ended as possible, we appreciate that some wording may have lacked clarity and thus the interpretation of results may be subjective. Similarly, interviewees were not asked identical questions, as each interview was shaped by the interviewee. Furthermore, some respondents of the survey believed that our research was being conducted prematurely. One respondent wrote: ‘I write about the COVID SAGE in the present tense because this is not over by a long way and I just think it's far too early to be doing stuff like this’, with another adding that ‘I'm feeling quite fatigued by the whole process at the moment and would need some more temporal distance from the whole process to be more objective about the interactions between modelling and policymaking’. We acknowledge the extreme pressure that scientists and advisers have been under since the beginning of the outbreak in Wuhan and understand that the views given at this stage of the pandemic may change in the future.

The effects of the COVID-19 pandemic are likely to define many years to come. The loss of life and destruction of livelihoods across the world, either directly or indirectly attributable to COVID-19, is substantial and must never be omitted from any discussion of the pandemic. Scientists and decision-makers have been placed in an extraordinarily challenging situation. As the pandemic continues, we are reminded of the responsibilities of both parties with respect to mathematical modelling and public health. Scientists have to ensure that their models are of the highest scientific quality while also acknowledging their inherent limitations and the urgent need for results, and decision-makers have to understand the nuances underlying results and consider outputs in conjunction with all other evidence. We hope that our article contributes to an important discussion of the interplay between these parties and highlights matters to consider ahead of the next emergency.

## Data accessibility

Electronic supplementary material, appendix S1 provides the quantitative survey results. Written answers to survey questions and transcripts of interviews are not provided to protect the confidentiality of participants. Answers to the multiple-choice questions are provided in the electronic supplementary material [96].
